# Abandoning the National Early Warning Score in our district general hospital

**DOI:** 10.1186/cc13251

**Published:** 2014-03-17

**Authors:** B Mylrea Lowndes, M Mercer, H Robinson

**Affiliations:** 1 Torbay Hospital, Torquay, UK

## Introduction

Early Warning Scores (EWS) are used in UK hospitals to identify patients who are acutely unwell or needing urgent review. The National EWS (NEWS) [1] was implemented in our institution and led to a noticeable increase in the numbers of patients being triggered for escalation of medical care, although clinically this was thought unwarranted. This led to a potentially dangerous lack of faith in the NEWS by clinical staff. We assessed whether it was safe to move to VitalPAC™ EWS (ViEWS), a commercially available electronic EWS [2]

## Methods

All patients scored as 'high risk' by NEWS (with a score of 6 or more) in a snapshot audit of patients in our 500-bed acute district general hospital were identified and reviewed clinically. All of these patients were then recategorised using ViEWS. The clinical safety of this recategorisation was then assessed.

## Results

Forty-six patients were identified in our hospital at the time of the snapshot as being high risk according to NEWS. After recategorising this cohort of patients using ViEWS, 36 were classified as high risk (in this instance meaning a score of 5 or more). Subjectively the authors did not have any clinical concerns created by moving 10 patients out of the high-risk classification.

## Conclusion

ViEWS is more specific without being less sensitive. We have replaced NEWS with ViEWS and feel that this is clinically safe.
